# Supplementary material for: MXene Based Nanocomposites for Recent Solar Energy Technologies
Source: Nanomaterials (Basel). 2022 Oct 18;12(20):3666. doi: 10.3390/nano12203666 (PMC9609812; doi:10.3390/nano12203666)
Supplement: Supplementary file 1 [file nanomaterials-12-03666-s001.zip › nanomaterials-1944409-supplementary.pdf]

nanomaterials

MDPI

Supplementary Materials

MXene Based Nanocomposites for Recent Solar Energy Technologies

T. F. Alhamada [1,2], M. A. Azmah Hanim [2,3,*], D. W. Jung [4,*], R. Saidur [5], A. Nuraini [2] and W. Z. Wan Hasan [6]

1 Department of Scientific Affairs, University Presidency, Northern Technical University, Mosul 41001, Iraq
2 Department of Mechanical and Manufacturing Engineering, Faculty of Engineering, Universiti Putra Malaysia, Serdang 43400, Selangor, Malaysia
3 Advance Engineering Materials and Composites Research Center (AEMC), Faculty of Engineering, Universiti Putra Malaysia, Serdang 43400, Selangor, Malaysia
4 Department of Mechanical Engineering, Jeju National University, 1 Ara 1-dong, Jeju 690-756, Korea
5 Centre for Nano-Materials and Energy Technology (RCNMET), School of Engineering and Technology, Sunway University, Petaling Jaya 47500, Selangor, Malaysia
6 Department of Electrical and Electronic Engineering, Faculty of Engineering, UPM, Serdang 43400, Selangor, Malaysia
* Correspondence: azmah@upm.edu.my (M.A.A.H.); jungdw77@naver.com (D.W.J.)

Table S1. Summary of the key parameters for the solar cells employing MXenes.

**Device** characterisation	FF (%)	Voc (V)	Jsc (mA·cm^{-2})	PCE (%)	Year	Ref.
MXene/m-SWCNTs (2:1)	80	1.073	25.09	21.42	2021	[1]
ITO/ETL/$CH_3NH_3PbI_3$/spiro-OMeTAD/Ag	74	1.07	23.11	18.29	2021	[2]
ITO/SnO_2/perovskite/spiro-MeOTAD/MoO_3/Au	0.798	1.14	24.96	23.30	2021	[3]
FTO/SnO_2/$CH_3NH_3PbI_3$/spiro-OMeTAD/Au	73.66	1.12	23.48	19.27	2021	[4]
FTO/SnO_2-$TiOxCl_{4-2x}$/$CsPbBr_3$:$Ti_3C_2Cl_x$/$Ti_3C_2Cl_x$/carbon	82.7	1.702	7.87	11.08	2021	[5]
ITO/SnO_2-Ti_3C_2/$MAPbI_3$/Spiro-OMeTAD/Ag	75	1.06	23.14	18.34	2019	[6]
ITO/ZnO:$Ti_3C_2T_x$/PTB7:$PC_{71}BM$ /MoO_3/Ag	69.33	0.77	17.53	09.36	2020	[7]
(ITO/treated Nb_2CT_x/$MAPbI_3$)	79.75	1.128	23.06	20.74	2021	[8]

Table S2. Summary of the key parameters for the solar cells employing metal oxide.

**Device** characterisation	FF (%)	Voc (V)	Jsc (mA·cm^{-2})	PCE (%)	Year	Ref.
Glass/ITO/Au:TiO2/PBDTTT-C-T:PC71BM/MoO3	64.30	0.78	16.21	08.20	2013	[9]
Glass/ITO/TiOx/brookite TiO2/CH3NH3PbI3/spiro-OMeTAD/Au	79.00	1.14	22.5	20.02	2018	[10]
Glass/ITO/GZO-ZnO/PffBT4T-2OD:PC70BM/MoOx/Al	67.6	0.77	18.6	09.74	2017	[11]
Glass/FTO/TiO2-NP/ZnO-NS: D-149/Pt	62.8	0.57	19.5	07.07	2011	[12]

Table S3. Summary of the key parameters for the solar cells employing noble metals.

**Device** characterisation	FF (%)	Voc (V)	Jsc (mA·cm^{-2})	PCE (%)	Year	Ref.
Glass/ITO/PEDOT:PSS:Au@AgNCs/PTB7: PC70BM/TiOx/Al	68.00	0.74	17.38	8.74	2014	[13]
Glass/ITO/PEOT:PSS/p-DTS(FBTTh2)2:PC70BM:Au-SiO2NR/Ca/Ag	70.04	0.77	15.40	8.20	2015	[14]
Glass/ITO/PEDOT:PSS:AgNP-67/PTB7:PC70BM/TiOx/Al	70.00	0.75	16.33	8.60	2013	[15]
Glass/ITO/PEDOT:PSS/PNP-535(front)/PIDTT-DFBT:PC71BM/C60-bis/ Ag	62.00	0.96	13.72	8.50	2014	[16]

References

1. Bati, A.S.R.; Hao, M.; Macdonald, T.J.; Batmunkh, M.; Yamauchi, Y.; Wang, L.; Shapter, J.G. 1D-2D Synergistic MXene-Nanotubes Hybrids for Efficient Perovskite Solar Cells. *Small* **2021**, *17*, 2101925. https://doi.org/10.1002/SMLL.202101925.
2. Yang, L.; Kan, D.; Dall'Agnese, C.; Dall'Agnese, Y.; Wang, B.; Jena, A.K.; Wei, Y.; Chen, G.; Wang, X.F.; Gogotsi, Y.; et al. Performance improvement of MXene-based perovskite solar cells upon property transition from metallic to semiconductive by oxidation of Ti3C2Tx in air. *J. Mater. Chem. A* **2021**, *9*, 5016–5025. https://doi.org/10.1039/D0TA11397B.
3. Yang, Y.; Lu, H.; Feng, S.; Yang, L.; Dong, H.; Wang, J.; Tian, C.; Li, L.; Lu, H.; Jeong, J.; et al. Modulation of perovskite crystallization processes towards highly efficient and stable perovskite solar cells with MXene quantum dot-modified SnO*Energy Environ. Sci.* **2021**, *14*, 3447–3454. https://doi.org/10.1039/D1EE00056J.
4. Zhao, Y.; Zhang, X.; Han, X.; Hou, C.; Wang, H.; Qi, J.; Li, Y.; Zhang, Q. Tuning the reactivity of PbI2 film via monolayer Ti3C2Tx MXene for two-step-processed CH3NH3PbI3 solar cells. *Chem. Eng. J.* **2021**, *417*, 127912. https://doi.org/10.1016/J.CEJ.2020.127912.
5. Zhou, Q.; Duan, J.; Du, J.; Guo, Q.; Zhang, Q.; Yang, X.; Duan, Y.; Tang, Q. Tailored Lattice "Tape" to Confine Tensile Interface for 11.08%-Efficiency All-Inorganic CsPbBr3 Perovskite Solar Cell with an Ultrahigh Voltage of 1.702 V. *Adv. Sci.* **2021**, *8*, 2101418. https://doi.org/10.1002/ADVS.202101418.
6. Yang, L.; Dall'Agnese, Y.; Hantanasirisakul, K.; Shuck, C.E.; Maleski, K.; Alhabeb, M.; Chen, G.; Gao, Y.; Sanehira, Y.; Jena, A.K.; et al. SnO2–Ti3C2 MXene electron transport layers for perovskite solar cells. *J. Mater. Chem. A* **2019**, *7*, 5635–5642. https://doi.org/10.1039/C8TA12140K.
7. Hou, C.; Yu, H. ZnO/Ti3C2Tx monolayer electron transport layers with enhanced conductivity for highly efficient inverted polymer solar cells. *Chem. Eng. J.* **2021**, *407*, 127192. https://doi.org/10.1016/J.CEJ.2020.127192.
8. Zhang, J.; Huang, C.; Yu, H. Modulate the work function of Nb2CTx MXene as the hole transport layer for perovskite solar cells. *Appl. Phys. Lett.* **2021**, *119*, 033506. https://doi.org/10.1063/5.0057978.
9. Xie, F.X.; Choy, W.C.H.; Sha, W.E.I.; Zhang, D.; Zhang, S.; Li, X.; Leung, C.W.; Hou, J. Enhanced charge extraction in organic solar cells through electron accumulation effects induced by metal nanoparticles. *Energy Environ. Sci.* **2013**, *6*, 3372–3379. https://doi.org/10.1039/C3EE42440E.
10. Kogo, A.; Sanehira, Y.; Numata, Y.; Ikegami, M.; Miyasaka, T. Amorphous Metal Oxide Blocking Layers for Highly Efficient Low-Temperature Brookite TiO2-Based Perovskite Solar Cells. *ACS Appl. Mater. Interfaces* **2018**, *10*, 2224–2229. https://doi.org/10.1021/ACSAMI.7B16662/SUPPL_FILE/AM7B16662_SI_001.PDF.
11. Sharma, R.; Lee, H.; Borse, K.; Gupta, V.; Joshi, A.G.; Yoo, S.; Gupta, D. Ga-doped ZnO as an electron transport layer for PffBT4T-2OD: PC70BM organic solar cells. *Org. Electron.* **2017**, *43*, 207–213. https://doi.org/10.1016/J.ORGEL.2017.01.028.
12. Lin, C.Y.; Lai, Y.H.; Chen, H.W.; Chen, J.G.; Kung, C.W.; Vittal, R.; Ho, K.C. Highly efficient dye-sensitized solar cell with a ZnO nanosheet-based photoanode. *Energy Environ. Sci.* **2011**, *4*, 3448–3455. https://doi.org/10.1039/C0EE00587H.
13. Baek, S.W.; Park, G.; Noh, J.; Cho, C.; Lee, C.H.; Seo, M.K.; Song, H.; Lee, J.Y. Au@Ag core-shell nanocubes for efficient plasmonic light scattering effect in low bandgap organic solar cells. *ACS Nano* **2014**, *8*, 3302–3312. https://doi.org/10.1021/NN500222Q/SUPPL_FILE/NN500222Q_SI_001.PDF.
14. Xu, X.; Kyaw, A.K.K.; Peng, B.; Xiong, Q.; Demir, H.V.; Wang, Y.; Wong, T.K.S.; Sun, X.W. Influence of gold-silica nanoparticles on the performance of small-molecule bulk heterojunction solar cells. *Org. Electron.* **2015**, *22*, 20–28. https://doi.org/10.1016/J.ORGEL.2015.03.026.
15. Baek, S.W.; Noh, J.; Lee, C.H.; Kim, B.; Seo, M.K.; Lee, J.Y. Plasmonic forward scattering effect in organic solar cells: A powerful optical engineering method. *Sci. Rep.* **2013**, *3*. https://doi.org/10.1038/SREP01726.
16. Yao, K.; Salvador, M.; Chueh, C.C.; Xin, X.K.; Xu, Y.X.; Dequilettes, D.W.; Hu, T.; Chen, Y.; Ginger, D.S.; Jen, A.K.Y. A General Route to Enhance Polymer Solar Cell Performance using Plasmonic Nanoprisms. *Adv. Energy Mater.* **2014**, *4*, 1400206. https://doi.org/10.1002/AENM.201400206.
